# A functional genomics screen reveals a strong synergistic effect between docetaxel and the mitotic gene DLGAP5 that is mediated by the androgen receptor

**DOI:** 10.1038/s41419-018-1115-7

**Published:** 2018-10-19

**Authors:** Kay Hewit, Emma Sandilands, Rafael Sanchez Martinez, Daniel James, Hing Y. Leung, David M. Bryant, Emma Shanks, Elke K. Markert

**Affiliations:** 1 Institute of Cancer Sciences, University of Glasgow, Glasgow, UK; 2 Scottish National Blood Transfusion Service, NSS, Glasgow, UK; 3 Cancer Research UK Beatson Institute, Glasgow, UK

## Abstract

Based on a molecular classification of prostate cancer using gene expression pathway signatures, we derived a set of 48 genes in critical pathways that significantly predicts clinical outcome in all tested patient cohorts. We tested these genes in a functional genomics screen in a panel of three prostate cancer cell lines (LNCaP, PC3, DU145), using RNA interference. The screen revealed several genes whose knockdown caused strong growth inhibition in all cell lines. Additionally, we tested the gene set in the presence of docetaxel to see whether any gene exhibited additive or synergistic effects with the drug. We observed a strong synergistic effect between DLGAP5 knockdown and docetaxel in the androgen-sensitive line LNCaP, but not in the two other androgen-independent lines. We then tested whether this effect was connected to androgen pathways and found that knockdown of the androgen receptor by si-RNA attenuated the synergy significantly. Similarly, androgen desensitized LNCaP-AI cells had a higher IC_50_ to docetaxel and did not exhibit the synergistic interaction. Short-term exposure to enzalutamide did not significantly alter the behaviour of parental LNCaP cells. An immunofluorescence analysis in LNCaP cells suggests that under the double insult of DLGAP5 knockdown and docetaxel, cells predominantly arrest in metaphase. In contrast, the knockdown of the androgen receptor by siRNA appears to assist cells to progress through metaphase in to anaphase, even in the presence of docetaxel. Our data suggest that DLGAP5 has a unique function in stabilizing spindle formation and surviving microtubule assault from docetaxel, in an androgen-regulated cell cycle system.

## Introduction

Prostate cancer is a common disease—the third most common cancer in males—that is characterized clinically by a wide diversity of outcomes. While a large fraction of patients has indolent, localized and manageable disease, there is a smaller subset of patients that suffer from aggressive forms with lethal metastatic potential. Until recently, initial treatments including surgery, radiation, androgen deprivation therapy (ADT), and anti-androgen therapy, were followed by chemotherapy once recurrence set in. After two large-scale clinical trials (CHAARTED, STAMPEDE) showed benefits for combined treatments in advanced tumours^1^, chemotherapy, commonly with the agent docetaxel (DCT), can now be used together with ADT as an initial treatment for higher-grade tumours. However, while the improved guidelines extend the life of patients with aggressive prostate cancer, there is still no cure for this disease. Furthermore, while a multitude of clinical trials is underway to test other therapeutic agents in prostate cancer, at the time of writing DCT remains the most widespread chemotherapy that patients receive and the only standard recommendation.

Here we aim to explore further options to target the aggressive, lethal form of prostate cancer. To this end, we make use of a molecular classification of prostate cancer based on gene expression data that we established previously^2^. This classification system identifies a subtype of highly aggressive tumours with poor outcomes, characterized by gene expression signatures for embryonic and induced pluripotent stem cells (ESC, iPSC), and for loss of function of the tumour suppressors PTEN and p53. This ESC|PTEN-|p53- subtype is opposed to a normal-like subtype with a good prognosis, defined by differentiation and functional PTEN and p53 pathway signatures. We hypothesize that the ESC|PTEN-|p53- subtype may contain molecular features that make these tumours both more prone to metastasis and more resistant to therapies. We selected genes highly enriched in the ESC|PTEN-|p53- subgroup relative to the normal-like subgroup across several patient data sets. From these we curated a small set of 48 genes that were also associated with p53 function, cell cycle mechanics or stemness. We then utilised a functional genomics screen to test these genes in three metastatic prostate cancer lines, with and without the addition of DCT. Data analysis aimed to identify genes whose knockdown would either significantly inhibit the growth of the cell lines in general, or whose knockdown would be synergistic with DCT.

## Results

### A 48 gene signature predicts aggressive prostate cancer

**Fig. 1:**
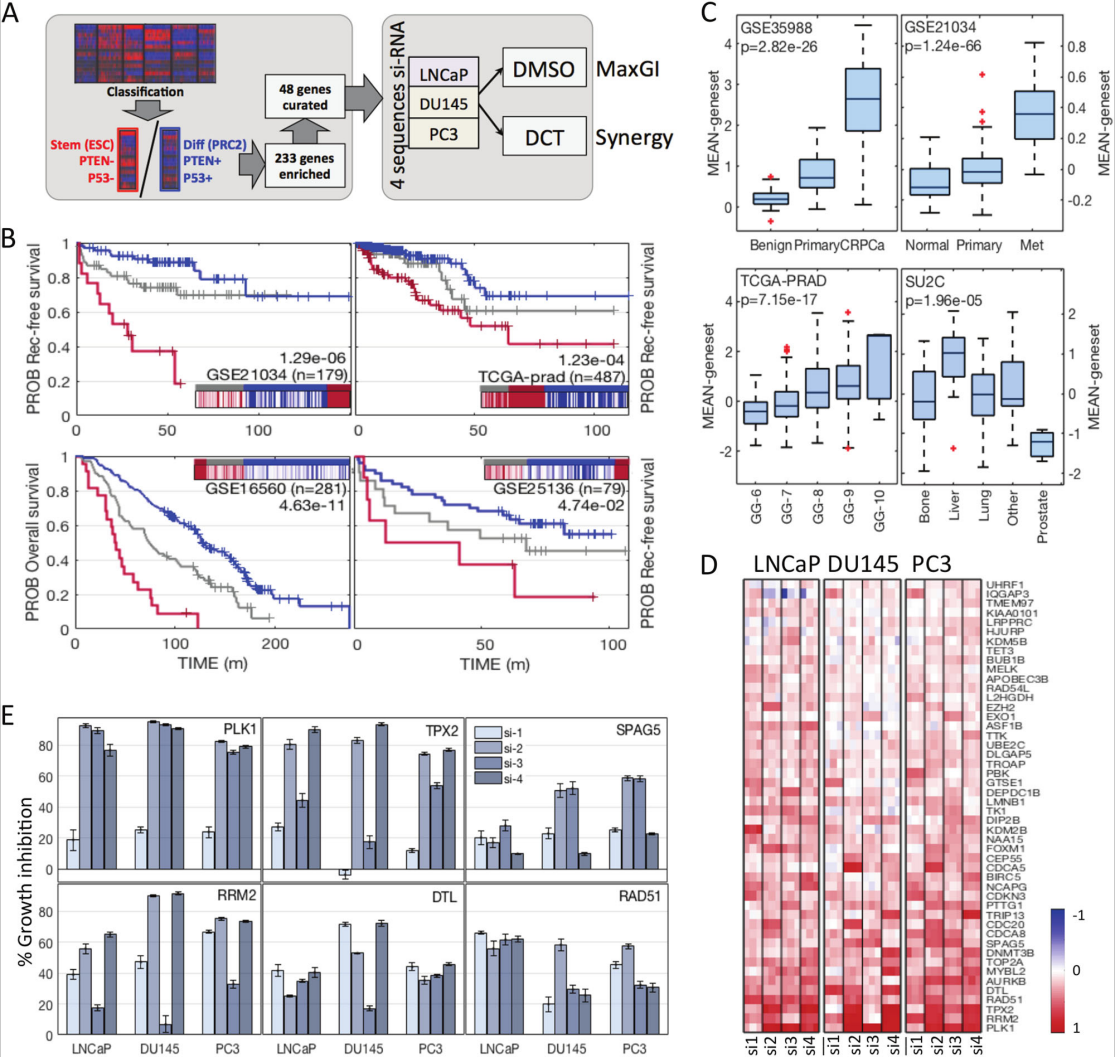
Functional genomics screen of 48 genes in aggressive prostate cancer **a** Schematic representation of gene selection and screen design. DCT, docetaxel. **b** The 48 gene set was evaluated for outcome prediction from (recurrence-free) survival data from four prostate cancer cohorts. Patient samples were clustered into three groups (kmeans) based on ssGSEA scores for the 48 gene set. Groups with high scores are shown in red, intermediate in grey and low in blue. Each plot is inset with a heatmap showing the clustering of scores. Logrank *p*-values (high versus low) are annotated in plots. **c** Mean expression of all 48 genes was used to plot differences by sample type. The gene set is highly expressed in castration-resistant prostate cancer (CRPCa, upper left), metastatic PCa (upper right), distant metastases (lower right), and high-Gleason PCa (lower left). Overall ANOVA *p*-values are annotated in the plots. **d** Heatmap illustrating screening results in DMSO condition. Columns are sorted into cell lines indicated on top, four individual siRNAs per gene (si-1/2/3/4) and three replicates. A full red colour indicates 100% growth inhibition relative to NTC, a blue colour indicates negative growth inhibition (GI). Genes are sorted top to bottom by increasing overall mean GI. **e** Average GI values for six genes of interest (mean of experiments + SEM, *n* = 3)

In order to determine genes that may affect outcomes in aggressive prostate cancer (PCa) we applied our previously developed classification scheme^2^ to data from three large PCa patient cohorts with associated survival outcomes (TCGA-PRAD, GSE21034, GSE16560)^3–5^. The classification scheme in particular detects an aggressive subtype that is characterized by the expression of pathway signatures indicating loss of PTEN or activation of the PI3K-AKT pathway, loss of p53 function, and stemness as indicated by loss of differentiation signals and gain of embryonic stem cell signatures (ESC|PTEN-|p53- subtype). The combination of these characteristic pathway enrichments effectively predicts malignant cancer and poor clinical outcome^2^. To collect these signatures into a more usable predictive gene set, we compared the ESC|PTEN-|p53- subtype in each cohort to the subtype with the opposite signature pattern, a ‘normal-like’ subgroup with a signature profile indicating differentiation and intact p53 and PTEN function. Differential expression analysis across all three sets produced a list of 233 genes most significantly enriched in ESC|PTEN-|p53- tumours versus normal-like tumours (see Methods, compare Fig. 1a). As expected, the list contained a large number of genes associated with cell proliferation. In order to focus our gene set on the characteristic pathways, we manually curated a subset of 48 genes using a keyword search on the extended GeneCards annotation, with keywords comprising terms such as ‘apoptosis’, ‘stem cell’ and ‘meiosis’ (see Methods). Proliferation genes were sub-selected for direct mechanistic function in the cell cycle and function within the PTEN-AKT/PI3K pathway. The final list is shown in Table 1. We evaluated the 48 gene list as a predictive signature on the three data sets and an additional patient data set with clinical annotation^6–9^, using single-sample gene set enrichment analysis (ssGSEA). The resulting scores for each data set were clustered into three groups (high, intermediate, low signature scores). A high score effectively predicted poor patient outcome in all sets (Fig. 1b). The gene set was also expressed at significantly higher levels in castration resistant prostate cancer (CRPCa) (Fig. 1c, upper left), and metastatic disease compared to primary PCa (Fig. 1c, upper right, Supp Fig. 1A, left), in distant metastatic samples compared to prostate recurrent disease (Fig. 1c, lower right, Supp Fig. 1A, right) and in high Gleason grade tumours (Fig. 1c, lower left), overall supporting the idea that these genes might contribute to metastatic potential, as well as treatment resistance (early recurrence, castration resistance). In addition, expression for all individual genes in the set was highly correlated in all data sets. This ensures in particular that individual genes in the list have a similar predictive value as the set (compare Table 2). We would like to point out here that the list of 48 genes was not derived as the most effective predictor of survival. Instead, we tried to focus on crucial pathways that might contribute directly and mechanistically to aggressive features.

**Table. 1:** The 48 gene list

Gene symbol	Entrez gene ID	Gene name
**APOBEC3B**	9582	Apolipoprotein B mRNA editing enzyme, catalytic polypeptide-like 3B
**ASF1B**	55723	Anti-silencing function 1B histone chaperone
**AURKB**	9212	Aurora kinase B
**BIRC5**	332	Baculoviral IAP repeat- containing 5
**BUB1B**	701	BUB1 mitotic checkpoint serine/threonine kinase B
**CDC20**	991	Cell division cycle 20
**CDCA5**	113130	Cell division cycle associated 5
**CDCA8**	55143	Cell division cycle associated 8
**CDKN3**	1033	Cyclin-dependent kinase inhibitor 3
**CEP55**	55165	Centrosomal protein 55 kDa
**DEPDC1B**	55789	DEP domain containing 1B
**DIP2B**	57609	DIP2 disco-interacting protein 2 homologue B (Drosophila)
**DLGAP5**	9787	Discs, large (Drosophila) homologue-associated protein 5
**DNMT3B**	1789	DNA (cytosine-5-)-methyltransferase 3 beta
**DTL**	51514	Denticleless E3 ubiquitin protein ligase homologue (Drosophila)
**EXO1**	9156	Exonuclease 1
**EZH2**	2146	Enhancer of zeste homologue 2 (Drosophila)
**FOXM1**	2305	Forkhead box M1
**GTSE1**	51512	G-2 and S-phase expressed 1
**HJURP**	55355	Holliday junction recognition protein
**IQGAP3**	128239	IQ motif containing GTPase activating protein 3
**KDM2B**	84678	Lysine (K)-specific demethylase 2B
**KDM5B**	10765	Lysine (K)-specific demethylase 5B
**KIAA0101**	9768	KIAA0101
**L2HGDH**	79944	L-2-hydroxyglutarate dehydrogenase
**LMNB1**	4001	Lamin B1
**LRPPRC**	10128	Leucine-rich pentatricopeptide repeat containing
**MELK**	9833	Maternal embryonic leucine zipper kinase
**MYBL2**	4605	v-myb avian myeloblastosis viral oncogene homologue-like 2
**NAA15**	80155	N(alpha)-acetyltransferase 15, NatA auxiliary subunit
**NCAPG**	64151	Non-SMC condensin I complex, subunit G
**PBK**	55872	PDZ binding kinase
**PLK1**	5347	Polo-like kinase 1
**PTTG1**	9232	Pituitary tumour-transforming 1
**RAD51**	5888	RAD51 recombinase
**RAD54L**	8438	RAD54-like (S. cerevisiae)
**RRM2**	6241	Ribonucleotide reductase M2
**SPAG5**	10615	Sperm associated antigen 5
**TET3**	200424	Tet methylcytosine dioxygenase 3
**TK1**	7083	Thymidine kinase 1, soluble
**TMEM97**	27346	Transmembrane protein 97
**TOP2A**	7153	Topoisomerase (DNA) II alpha 170 kDa
**TPX2**	22974	TPX2, microtubule-associated
**TRIP13**	9319	Thyroid hormone receptor interactor 13
**TROAP**	10024	Trophinin associated protein
**TTK**	7272	TTK protein kinase
**UBE2C**	11065	Ubiquitin-conjugating enzyme E2C
**UHRF1**	29128	Ubiquitin-like with PHD and ring finger domains 1

**Table 2:** Correlation and prognostic values of the 48 genes

Analysis	Correlation: 7 data sets			Survival: logrank high vs low, tertiles			
Gene	Mean PCC	Stderr PCC	Mean log_10_(PV)	TCGA	GSE21034	GSE16560	GSE25136
**APOBEC3B**	0.4856	0.0656	10.0992	1.71E-03	1.35E-01	2.04E-01	2.11E-02
**ASF1B**	0.8587	0.0325	42.3520	3.34E-04	1.25E-02	na	4.91E-01
**AURKB**	0.6929	0.0714	21.6256	5.58E-03	8.57E-03	na	3.59E-01
**BIRC5**	0.8350	0.0360	43.6592	3.33E-04	1.10E-03	7.25E-07	7.92E-02
**BUB1B**	0.8192	0.0593	47.0013	7.69E-04	1.76E-03	2.42E-02	1.54E-01
**CDC20**	0.7861	0.0787	37.6874	2.95E-05	4.01E-04	4.95E-02	1.18E-01
**CDCA5**	0.9218	0.0053	60.1906	3.67E-06	3.35E-03	na	na
**CDCA8**	0.8701	0.0486	50.5426	1.50E-04	6.42E-03	na	4.30E-01
**CDKN3**	0.8370	0.0317	43.7493	4.53E-03	5.89E-05	4.39E-04	6.98E-02
**CEP55**	0.8610	0.0418	48.5437	2.40E-03	2.92E-03	na	1.85E-01
**DEPDC1B**	0.8575	0.0280	49.0341	7.68E-03	5.62E-04	na	na
**DIP2B**	0.2916	0.0909	4.6882	4.87E-01	4.23E-01	na	na
**DLGAP5**	0.9002	0.0219	56.1288	4.81E-04	1.80E-04	na	2.65E-01
**DNMT3B**	0.6054	0.0372	15.7632	1.59E-03	9.30E-02	na	3.55E-01
**DTL**	0.8058	0.0435	42.5223	2.13E-03	3.76E-04	na	3.02E-01
**EXO1**	0.8281	0.0500	46.2383	2.23E-04	7.67E-03	na	2.84E-01
**EZH2**	0.7299	0.0609	28.9437	3.91E-04	8.41E-02	1.10E-02	7.81E-02
**FOXM1**	0.8335	0.0627	48.5116	1.13E-03	4.23E-04	3.88E-03	2.13E-01
**GTSE1**	0.6820	0.1217	26.8985	8.75E-05	1.08E-03	1.76E-01	1.25E-01
**HJURP**	0.7048	0.0814	20.2463	2.79E-04	9.97E-02	na	4.75E-01
**IQGAP3**	0.6107	0.1535	22.2494	2.42E-04	4.65E-04	na	na
**KDM2B**	0.4024	0.0749	9.5927	3.72E-01	2.97E-02	na	na
**KDM5B**	0.2053	0.1030	3.1869	1.40E-01	3.65E-01	na	9.14E-03
**KIAA0101**	0.7584	0.0641	32.3947	1.38E-03	4.84E-03	3.58E-03	7.48E-04
**L2HGDH**	0.2832	0.0595	3.3340	1.91E-01	1.80E-01	na	6.02E-02
**LMNB1**	0.7537	0.0679	35.3260	8.35E-04	8.12E-03	2.35E-03	2.89E-01
**LRPPRC**	0.1656	0.0661	2.0329	8.44E-03	2.58E-01	4.59E-02	7.25E-03
**MELK**	0.8342	0.0617	49.5800	3.21E-03	5.06E-05	3.22E-02	2.03E-04
**MYBL2**	0.7365	0.0773	32.5805	9.75E-06	2.61E-04	4.22E-02	1.45E-01
**NAA15**	0.2681	0.0687	3.6134	4.18E-01	na	na	7.01E-02
**NCAPG**	0.8956	0.0387	59.2214	1.68E-04	8.05E-04	na	1.48E-01
**PBK**	0.8279	0.0386	42.1333	4.35E-05	5.63E-05	na	1.57E-02
**PLK1**	0.7966	0.0774	42.1368	2.77E-06	3.71E-04	2.14E-01	1.84E-01
**PTTG1**	0.8281	0.0344	42.8207	1.68E-03	2.82E-03	9.37E-05	2.78E-02
**RAD51**	0.6930	0.0941	27.9681	1.44E-02	2.89E-02	3.45E-01	4.06E-01
**RAD54L**	0.7185	0.0951	29.8411	5.19E-05	1.06E-02	1.01E-01	4.39E-02
**RRM2**	0.8386	0.0362	44.2879	1.54E-04	7.28E-05	8.74E-04	9.17E-05
**SPAG5**	0.8269	0.0643	47.5110	2.63E-07	1.02E-03	1.57E-03	2.29E-01
**TET3**	0.3346	0.0705	3.8902	1.85E-01	3.58E-01	na	5.47E-02
**TK1**	0.8103	0.0325	37.8871	1.07E-04	3.49E-02	1.32E-03	4.63E-01
**TMEM97**	0.4398	0.0693	9.7922	8.43E-02	8.45E-02	4.25E-02	3.90E-01
**TOP2A**	0.8044	0.0493	37.9232	1.20E-04	9.33E-04	3.32E-04	1.32E-02
**TPX2**	0.8554	0.0752	56.1147	2.00E-06	3.63E-04	2.05E-01	3.11E-01
**TRIP13**	0.6271	0.1025	25.6237	1.23E-03	3.19E-02	2.17E-05	2.76E-01
**TROAP**	0.7652	0.0682	29.6392	2.06E-05	2.15E-02	7.45E-03	4.81E-01
**TTK**	0.6963	0.0593	29.1810	5.31E-03	1.58E-03	3.78E-01	2.38E-01
**UBE2C**	0.8870	0.0318	59.0475	5.03E-06	6.06E-04	3.86E-03	1.31E-01
**UHRF1**	0.8612	0.0150	43.4272	4.80E-05	2.91E-03	na	na

Pearson correlation coefficients (PCC) between individual gene expression and the mean expression of the 48 gene set were calculated in seven data sets (TCGA, GSE21034, GSE16560, GSE25136, GSE35988, SU2C, FHCC). Average PCC and standard errors are listed, along with the average –log10(*p*-value) of the correlations. Survival columns contain logrank *p*-values for Kaplan–Meier analyses for individual genes, calculated by splitting each data set into tertiles based on expression, and comparing high versus low tertiles. Note that in GSE25136 this yields lower *p*-values due to the small size of groups (*n* = 79)

### Functional genomics screen of 48 genes reveals novel growth inhibitors in aggressive PCa models

We tested the 48 genes in a functional genomics assay using siRNA knockdown in a panel of three PCa cell lines with aggressive features; LNCaP, PC3 and DU145 (Fig. 1a). Of these, only LNCaP cells are androgen sensitive with an intact active androgen receptor (AR), and wild-type p53. All three lines are defective in PTEN. The PC3 and DU145 lines served as models for metastatic disease, while LNCaP was included to model the difference towards androgen-sensitive disease. We performed a viability screen using commercially available sets of four individual siRNAs per gene (‘deconvoluted’ screen, cell count assay, see Methods). Deconvolution does not exclude false negative results, but may help pinpointing off-target effects. The screen was performed with two arms, where DCT or vehicle (DMSO) was added after 24 h. Data from the vehicle arm were evaluated for genes that affect growth across the three cell lines. A gene was considered significant if its knockdown caused growth inhibition (GI) above mean plus standard deviation in at least two sequences and in at least two cell lines (Fig. 1d, e). si-*PLK1* served as a positive control and was the top hit with GI > 78% in more than two sequences in all cell lines (PLK1 satisfied significance requirements in our human data analysis; its knockdown also inhibits growth effectively in many cancer cell lines^10,11^). The top two most effective growth inhibitory genes identified in the screen were *TPX2* ( > 70% GI, 2+ siRNAs) and *RRM2* ( > 55% GI, 2+ siRNAs, Fig. 1e). Both are unsurprising candidates as drivers of growth, since they are essential in mitosis, and in DNA duplication, respectively. Interestingly however, knockdown of Denticleless E3 Ubiquitin Protein Ligase Homologue (*DTL*) also showed good results in all cell lines with a GI > 40% in 2+ sequences (Fig. 1e, panel centre bottom). DTL is part of the DCX (DDB1-CUL4-X-box) complex that is required for cell cycle control, and particularly for DNA damage response and repair. DTL has been implicated in gastric tumour growth and invasion, and in ovarian cancers^12,13^. Furthermore, knockdown of sperm associated antigen 5 (*SPAG5*), a gene associated with spermatogenesis and mitosis regulation, had a pronounced effect in PC3 and DU145 cells, demonstrating GI > 50% in both with at least two siRNAs: it was less effective in LNCaP cells by a factor two (Fig. 1e, panel top right). This result is supported by a recent animal study showing that SPAG5 is upregulated in metastatic but not primary prostate tumours, and that its knockdown reduces tumour growth and metastasis in vivo^14^. Based on our results, one might speculate that this SPAG5 activity is linked with either the androgen status of the tumour cells, or their p53 status. Conversely *RAD51* knockdown produced a GI of 60% in LNCaPs, with approximately half that in the other cell lines (Fig. 1e, panel bottom right). RAD51 is a DNA damage response gene that has been associated with resistance to radiation and PARP inhibitors in PCa, and is co-regulated by p53^15–17^. Overall, the GI assay in DMSO detected both known PCa associated genes, as well as an interesting candidate not previously associated with PCa (*DTL*).

### Functional genomics screen in the presence of DCT reveals cell cycle genes *CDC20, TPX2* and *DLGAP5*

**Fig. 2:**
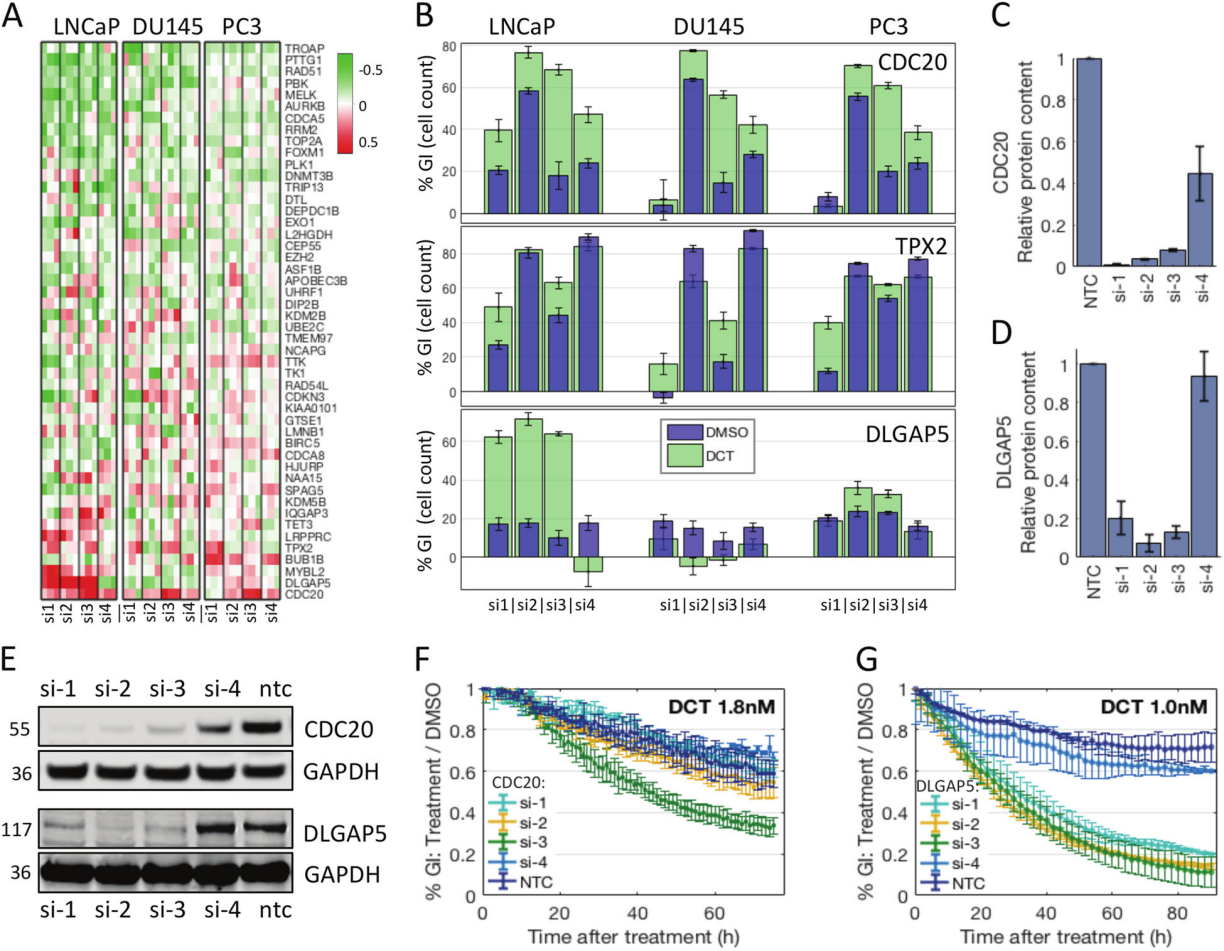
Results from the synergy screen with docetaxel. **a** Heatmap illustrating the results from the docetaxel (DCT) synergy screen. Columns are sorted as before. The colours show %GI under DCT relative to DMSO (window). A full red colour indicates a positive window of 50 percentage points, a green colour indicates a negative window. **b** Individual data for *CDC20*, *TPX2* and *DLGAP5*. In blue, growth inhibition in DMSO, relative to NTC (four siRNAs, three cell lines; shown is mean of experiments + SEM, *n* = 3). In green, growth inhibition in DCT relative to NTC in DCT. **c**, **d**, **e** Knockdown validation by western blot, performed in PC3 cells for the 4 individual siRNAs targeting *CDC20* (**c**, **e** top panels) and performed in LNCaP cells for the siRNAs targeting *DLGAP5* (**d**, **e** bottom panels). **f**, **g** Validation of growth inhibition by Incucyte growth assay. Growth was measured by area of confluence, values shown are treatment condition relative to DMSO condition, mean of experiments + SEM (*n* = 2). CDC20 validation was performed in PC3 cells (**f**), DLGAP5 validation was performed in LNCaP cells (**g**)

The 48 genes in the signature are highly expressed in metastatic and recurrent tumours (Fig. 1c). These tumour types are candidates for chemotherapy in the clinic. We therefore sought to test whether knockdown of any of these genes added to the effect of treatment with DCT, the standard chemotherapeutic agent in PCa and CRPCa. DCT was given at EC_30_ in each cell line (LNCaP, 1.8 nM, PC3, 1.36 nM, DU145, 3.28 nM) for 48 h. The results showed a moderate effect overall, with little difference in GI observed ±DCT (Mean %GI all genes, LNCaP, +DCT = 24.6%, −DCT = 19.8%: PC3, +DCT = 25.2%, −DCT = 24.2%: DU145, +DCT = 14.2%, −DCT = 23.1%). The effect of gene knockdown in vehicle treated cells was subtracted from those treated with DCT to generate a ‘window’ of efficacy. Again, little difference in overall window was observed between cell lines (mean + sd, all genes LNCaP −4.80 + 16.71, DU145 -3.54 + 11.97, PC3 −1.02 + 9.57, Fig. 2a). To identify candidates, genes with a window ≥ mean + sd in 2+ cell lines and 2+ siRNAs were selected: these were *CDC20, TPX2, MYBL2, DLGAP5, SPAG5*, and *LMNB1*. Of these, *CDC20* and *TPX2* satisfied the criteria in all cell lines. Strikingly, five of these six genes are directly involved in mitosis. Knockdown of *CDC20* had a window of GI > 10% in all cell lines and three siRNAs (Fig. 2b, top panel). CDC20 is part of the anaphase-promoting complex APC(CDC20), and recent reports corroborate synergistic effects with DCT in PCa^18,19^. We confirmed the knockdown capacity of the siRNAs by western blotting (Fig. 2c, e). Comparison with GI in DMSO condition (Fig. 2b) suggested off-target effects in some of the siRNAs. A validation growth assay performed in PC3 cells (Incucyte assay evaluating confluency of cell populations over time, see Methods) showed that one siRNA had a significant added effect with DCT (Fig. 2f). *TPX2* knockdown also had an added effect with DCT in all cell lines, in two siRNAs (Fig. 2b middle panel). TPX2 binds with AURKA and is expressed during mitosis, on the spindle microtubules emanating from the poles^20–22^. Its expression along these forms a gradient with the expression of another gene in the list, DLG associated protein 5 (DLGAP5), which is expressed on the chromatin side. The data for DLGAP5 suggested a strong synergistic effect with DCT that only occurred in LNCaP cells (Fig. 2b bottom panel). Because of the striking nature of this result, we focused on investigating DLGAP5.

### DLGAP5 knockdown is synergistic with DCT in LNCaP cells

We observed a strong synergistic effect between the knockdown of *DLGAP5* and DCT in LNCaP cells, but not in PC3 or DU145 cells. This synergistic effect was observed with three out of four individual siRNA sequences (combination index by Bliss independence model, CI < 0.635, stderr < 0.035, where CI < 1 signifies synergy, see Supp Fig. 1A, B), each of which achieved a window > 45 percentage points, and resulted in > 60% total GI in the presence of DCT (Fig. 2b bottom panel). This is a near 5-fold change of GI (*p* < 0.001), compared to a modest 1.3-fold change in PC3 and negative effect observed in DU145 cells. Consistently, the three effective siRNAs each produced an >80% knockdown of relative protein content by western blotting, while the fourth was ineffective (Fig. 2d, e bottom panels). We further confirmed the synergistic effect using an Incucyte growth assay in LNCaP cells with different doses of DCT (Fig. 2g, Supp Fig. 2C). The same three siRNAs were effective in this assay. While the knockdown alone did not affect growth significantly, we observed a dramatic growth reduction with addition of DCT at 1 nM, producing a significant divergence of relative growth curves (*p* < 0.01, by AUC). Phenotypically, the cells appeared to undergo growth arrest followed by apoptosis (rounding, blebbing, Supp Fig. 1E). In PC3 cells, the Incucyte growth assay did not reveal significant added effects (0.5–3 nM DCT, data not shown). For the remainder of the study we used a pool of the three effective siRNAs, referred to as siDLGAP5.

### DLGAP5-DCT synergy is attenuated by loss of AR function

**Fig. 3:**
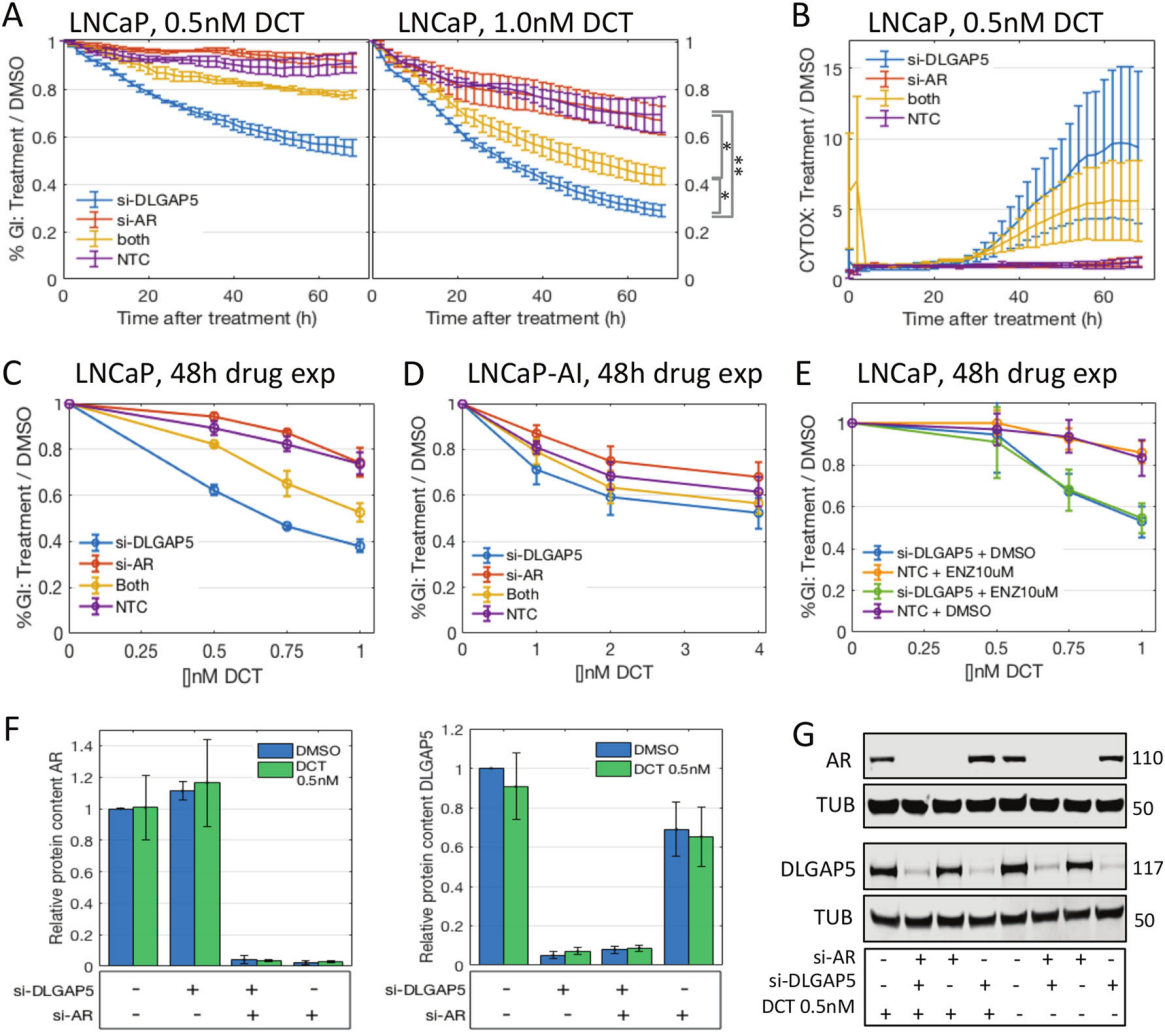
DLGAP5 knockdown synergizes with DCT in an androgen-dependent manner. **a** Incucyte growth assay: Growth was measured as area of confluency. Curves show growth inhibition of DCT treatment relative to DMSO, for two doses of DCT. Shown is mean of experiments + SEM (*n* = 3). *DLGAP5* knockdown (pool of three siRNAs, blue curves) synergizes with DCT in this assay. The effect is attenuated by additional *AR* knockdown (pool of four siRNAs, yellow curves). Significance was calculated by comparing AUC followed by T-test (shown for 1.0 nM condition, right hand side). *p < 0.05, **p < 0.01. **b** Toxicity assay with CytoxGreen shows a similar attenuation under AR knockdown. **c** Dose response curves at 48 h drug exposure. **d** Dose response curves in androgen desensitized LNCaP-AI cells. In doses up to 4 nM DCT there is no significant effect of DLGAP5. **e** Dose response curves in parental LNCaP cells that were exposed to androgen inhibition by enzalutamide for 1 h prior to DCT exposure show no attenuation. **f**, **g** Western blots for DLGAP5 and AR protein confirm the knockdown effect of the siRNA pools, and show no significant difference of DLGAP5 and AR protein under AR and DLGAP5 knockdown, respectively

In contrast to PC3 and DU145, LNCaP cells are androgen-sensitive. We therefore tested whether the synergistic effect would change under AR knockdown. Combining siDLGAP5 and siAR attenuated the synergy between DCT and DLGAP5 knockdown (Fig. 3a, significance measured by AUC). This was seen both in the confluency growth assays and a CytoxGreen cytotoxicity assay performed in the Incucyte (Fig. 3b). Knockdown of AR alone did not have a significant effect on DCT sensitivity, while DLGAP5 knockdown shifted the EC_30_ into the sub-nanomolar range ( < 0.5 nM, 48 h DCT exposure, Fig. 3c), with intermediate values for the double knockdown (0.5 < EC_30_ < 1.0 nM). We then tested an AR independent sub-line, LNCaP-AI, that was cultured in charcoal-stripped hormone free medium. LNCaP-AI cells were more resistant to DCT than LNCaPs, with a lower response rate at higher doses (EC_40_ > 4 nM compared to < 2 nM in LNCaPs). Remarkably, the LNCaP-AI cells did not respond synergistically to siDLGAP5 + DCT in this dose range (Fig. 3d). On the other hand, short-term suppression of AR activity with the AR antagonist enzalutamide did not change the synergy effect of siDLGAP5 + DCT in LNCaPs (Fig. 3e). Similarly, switching LNCAP-AI cells to regular medium supplemented with 10 nM dihydrotestosterone (DHT) at transfection did not reproduce the effect seen in the parental cells (Supp Fig. 2B). We then tested whether AR knockdown had an effect on DLGAP5 protein expression and vice versa, with and without DCT (Fig. 3f, g). There was no significant difference in protein levels relative to control in either case. Furthermore, DHT stimulation of LNCaPs increased DLGAP5 protein levels only slightly (*p* > 0.1), consistent with a small increase in mitotic rate (23.5%, measured by CCNB1 levels, *p* > 0.1, Supp Fig. 2C, D). Microarray data sets describing LNCaP cells stimulated by 24 h of 10 nM DHT (GSE60721, GSE4636, GSE69330^23–25^) confirmed a slight increase of both DLGAP5 and CCNB1 (Supp Fig. 2E). Taken together these data support an indirect interaction between DLGAP5 and AR, rather than a direct one.

### DLGAP5-DCT synergy impacts metaphase, attenuated by AR knockdown

**Fig. 4:**
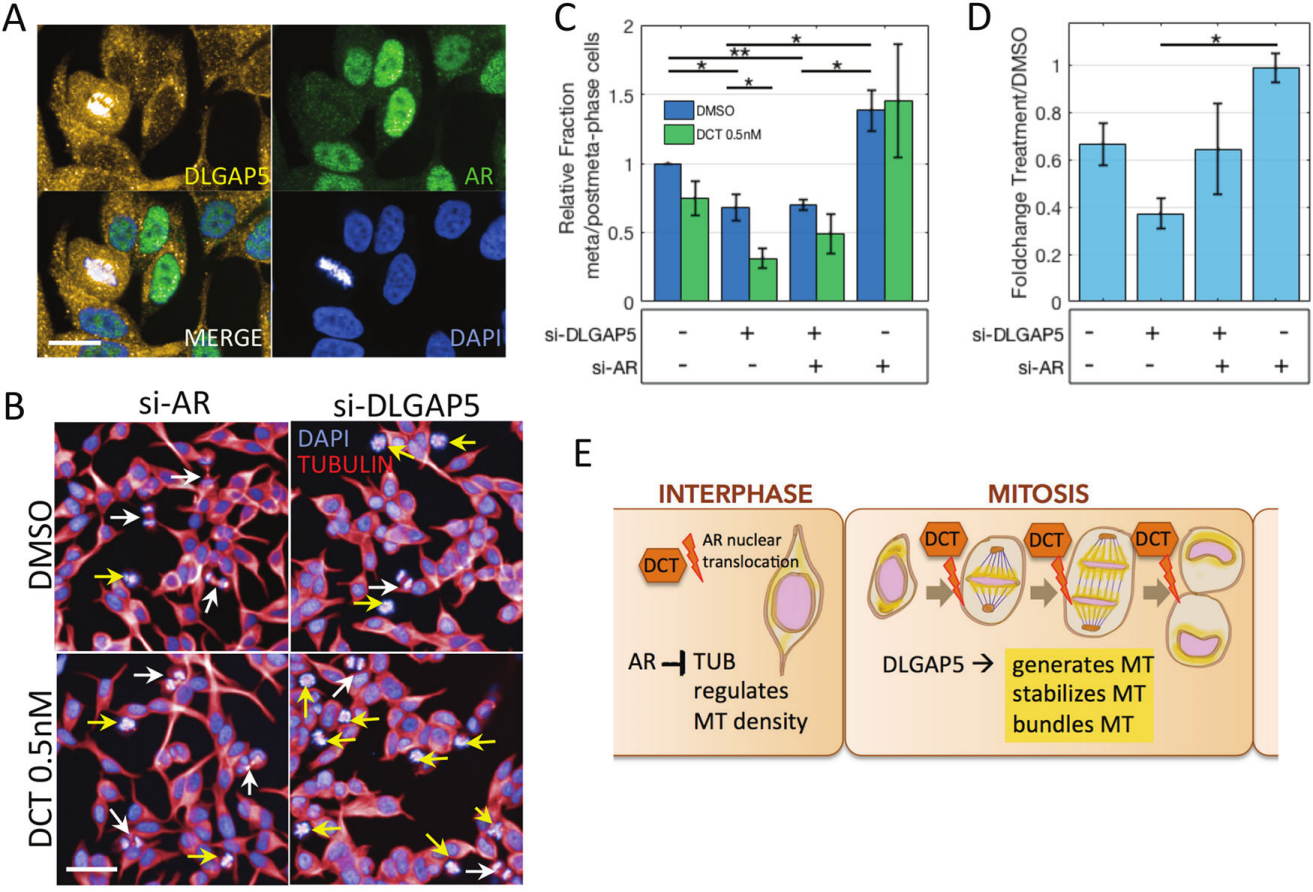
Androgen receptor knockdown might support transition of cells to anaphase phase, counteracting the DLGAP5 and DCT-induced halt in metaphase. **a** Immunofluorescence analysis of LNCaP cells transfected with NTC siRNA show the characteristic expression of DLGAP5 on the kinetochore side of the mitotic spindle, while AR protein is depleted during mitosis. Scale bar, 20 μm. **b** Fields of LNCaP cells with DLGAP5 and AR knockdown, respectively, with and without the addition of 0.5 nM DCT (12 h). AR knockdown led to a higher fraction of post-metaphase mitotic cells (white arrows), in contrast to DLGAP5 knockdown, which led to a higher fraction of metaphase mitotic cells (yellow arrows) in DMSO, and a further increase of metaphase cells in DCT. Scale bar, 50 μm. **c** Quantification of counts ratios, post-metaphase relative to metaphase cells. Mean of experiments + SEM (*n* = 3). *p < 0.05, **p < 0.01. **d** Change of the post-metaphase/metaphase ratios in DCT versus DMSO in the different siRNA conditions. Mean of experiments + SEM (*n* = 2). *p < 0.05. **e** Schematic illustration of a proposed interaction between AR, DLGAP5 and DCT. In AR sensitive cells, androgen might down-regulate tubulin expression, which in turn could affect the amount of microtubule (MT) carried over from interphase into metaphase. During mitosis, DLGAP5 contributes to MT generation at the kinetochores and supports MT organization. DLGAP5 is expressed strongly at the chromatin side of the spindle MT during meta-and anaphase (shaded in yellow). DCT interferes with MT dynamics during this phase. Loss of DLGAP5 and an active AR system might both contribute to lower spindle density and weaker MT organisation, producing higher sensitivity to DCT

Direct interaction between the AR and DLGAP5 proteins is indeed unlikely given their expression patterns during the cell cycle. DLGAP5 is strongly expressed during all phases of mitosis, is located at the spindle microtubule^26,27^, and is present at low levels during the remainder of the cell cycle^28^. The AR protein is present and builds up during the cycle but vanishes at the onset of mitosis where it remains absent until division is complete^29^. We confirmed this effect in our cells, using an ICC/IF assay to analyse mitotic health (Fig. 4a). The degradation of the AR during mitosis was unaffected by DLGAP5 knockdown (Supp Fig. 2F). Likewise, AR knockdown did not seem to suppress expression of DLGAP5 at the mitotic spindle apparatus (not shown). DLGAP5 is involved in microtubule organisation during mitosis^27,30^. It is expressed on the kinetochores of spindle fibres and has been implicated in the nucleation of central spindle microtubules during anaphase^28^. The gradient it forms with TPX2 towards the spindle poles during mitosis might also be involved in the contraction of spindle microtubule in anaphase^21^. This indicates that the metaphase-to-anaphase transition might be critical in our setting. DCT arrests cells in metaphase, and consistently we found that under DCT (0.5 nM), the ratio of metaphase-to-post-metaphase mitotic cells was decreased (Fig. 4b, c). This ratio decreased even further under siDLGAP5 + DCT (0.6FC to 0.4FC, Fig. 4d), suggesting that the cells are predominantly arrested in metaphase in this condition. Interestingly, the ratio was increased in siAR treated cells (Fig. 4c), and was not significantly changed by DCT in that condition (Fig. 4d). This indicates that AR knockdown might help cells progress through metaphase and initiate anaphase under DCT insult.

## Discussion

Recent large-scale clinical trials (STAMPEDE, CHAARTED) have shown that advanced prostate cancers respond better to a first-line combination of chemotherapy (DCT) and ADT than to the sequential treatment where ADT is given first and chemotherapy is given upon recurrence. However, while the clinical benefits of earlier chemotherapeutic intervention are clear from these trials^1^, advanced prostate cancer is still a lethal disease and further improvements are urgently needed. The trial data seems to suggest earlier, more aggressive intervention for the most aggressive tumours; this would suggest an early point of action also for future targeted therapies and novel combination approaches. The data also emphasizes the importance of androgen status for treatment efficacy and supports treating ADT-naïve tumours with complementary (i.e. non-hormone) therapy. Here we have analysed a set of 48 genes that were selected based on their expression and potential function in a highly aggressive, molecularly defined subgroup of human cancer samples. We have evaluated knockdown of these genes in models of advanced PCa including both androgen-independent and -sensitive cell lines. Especially in the above context, the present results suggest an interesting novel interaction between the androgen receptor system and mitotic dynamics under DCT. We have shown here that loss of the mitotic gene DLGAP5 acutely sensitizes androgen-dependent LNCaP cells to DCT treatment. This effect is attenuated by knockdown of the AR, and is absent in the androgen-independent LNCaP-AI cell line variant, as well as in androgen-independent PC3 and DU145 cells.

However, the effect is not attenuated under short-term use of the AR antagonist enzalutamide and it is not re-established in LNCaP-AI under acute exposure to hormone. This suggests that only full transcriptional loss of the AR, or long-term transformation to androgen independence affect the DLGAP5-DCT synergy. We note here that, while LNCaP-AI cells do express the AR, its downstream transcriptional activity is altered as the cells become androgen independent and this affects in particular also cell cycle regulation^31–33^. This in turn seems to suggest that cells with an active and well-established androgen pathway rely on DLGAP5 to stabilize mitotic health and function, while androgen-independent cells do not nearly as much.

Our immunofluorescence studies suggest that the critical phase of mitosis is the transition from meta- to anaphase. Based on this data, the formation of central spindle microtubule might be the point of vulnerability. Others have shown that a considerable fraction of central spindle microtubule is formed by DLGAP5, while another part is recruited from remaining interphase microtubule^28^. This latter part could be affected in androgen-dependent cells, as androgen can suppress tubulin expression and might affect spindle density^34^ and microtubule nucleation at the centrosomes during interphase^35^. Thus androgen-dependent cells without DLGAP5 might be increasingly vulnerable to DCT simply because the density of microtubule in their spindles and especially their central spindles, is lower, requiring a lower molecule content of drug to bind and stabilize the spindle microtubule. In other words, in these cells, DLGAP5 might make up for a lack of ambient microtubule to create sufficiently thick spindle fibre bundles (Fig. 4e). We are currently expanding our ICC/IF studies to analyse spindle density. DLGAP5 also has binding sites for several androgen-regulated genes in its promoter region, including most prominently SREBF1 and NCOR1, which have been linked to cell-cycle regulation^36–38^. We are currently investigating these potential regulatory interactions.

This interaction between the androgen-regulated cell cycle, DCT and DLGAP5 might also extend to other cancers. As reported previously^39^, DLGAP5 levels alone have predictive power in prostate cancer (Table 2). However, the gene was first found as a prognostic indicator in hepatocellular carcinoma (HCC)^40,41^. HCC is more common in males than in females (2.89-fold higher incidence, 2.47-fold higher mortality, SEER 18 2011–2015, https://seer.cancer.gov/statfacts/html/livibd.html), and numerous studies have investigated the AR pathway in this disease^42–47^. In fact, DLGAP5 gene expression very effectively predicts outcome in male HCC patients, but much less so in females (Supp Fig 3A, B, C). DLGAP5 expression is not gender-biased in these tumours (TCGA-LIHC, *p* = 0.52, *n* = 115 female, *n* = 239 male), suggesting that interaction networks and background might play a role in the gene’s functionality. The gene is also a predictor of poor outcome in ER-positive breast cancers, but not in ER-negative, or HER2-positive ones (Supp Fig 3D, E, F), indicating again a connection with hormone status.

Overall, here we show a novel feature of androgen-sensitive prostate cancer cells compared to independent or desensitized ones that might provide a potential new synergistic target for androgen-sensitive, aggressive prostate cancers that qualify for first-line DCT treatment.

## Methods

### Cell culture

LNCaP, DU145 and PC3 were cultured in RPMI 1640 medium supplemented with glutamine and 10% fetal bovine serum. LNCaP-AI cells were cultured in charcoal-stripped DMEM medium minus phenol red.

### siRNA transfection and screen

Human OnTarget plus siRNAs were ordered from Horizon Discovery (Dharmacon), either individual (screening library) or as pool (AR). Cells were transfected with siRNAs using a reverse transfection protocol with Lipofectamine RNAiMAX (Invitrogen), following the manufacturer’s instructions. The same protocol was used for all cell lines transfected with siRNA.

### Viability screen

SiRNA screening was performed in triplicate with a custom library of 48 human genes, and a set of four individual siRNAs per gene (Horizon). Cell stocks were grown to confluency, harvested, counted and seeded in 96-well plates at the following densities to reflect differing growth rates: PC3 at 3000 cells per well (cpw), DU145 at 2500 cpw and LNCaP at 5000 cpw. A reverse transfection method was used in order to transfect cells with siRNA. Subsequently cell suspensions were added to the plates using an XRD automated reagent dispenser (FluidX). Plates were incubated at 37 °C, 5% CO2 for 24 h, after which either EC_30_ DCT (PC3: 1.36 nM), (DU145: 3.28 nM), (LNCaP: 1.80 nM) or DMSO was added to cells. After incubation for a further 48 h, the medium was removed and cells were fixed with 4% formaldehyde and stained with DAPI/Tx-100 in 1x PBS (0.25 µg/ml/0.001% final concentration/well). Images of nuclei were acquired using the Operetta High Content Imaging system (PerkinElmer) and the number of nuclei in each well were quantified as a measure of cell growth using Columbus High Content Imaging and Analysis Software (PerkinElmer).

### Incucyte growth assay

Cells were seeded in 96-well Greiner black glass bottom plates (Greiner 665090), and transfected with siRNA at seeding where needed. Depending on cell type, wells were seeded with 5000 to 12000cpw in 100 ul medium. Plates were incubated overnight and transferred to the Incucyte. Drugs were added with additional 50 ul medium (spike-in) between scans. CytoxGreen reagent was added according to manufacturer’s protocol along with drugs, where required. In each experiment, plates contained three or more replicate wells per condition and two pictures were taken per well per scan. Confluence data were downloaded, plotted and analysed using MATLAB custom routines. In particular, for relative GI, we first normalized confluence growth curves by the confluence at time of drug addition in order to balance out variation in seeding. We then calculated the ratios of confluence measures for drug over DMSO for each condition, in each experiment. We then plotted the mean of experiments with the SEM indicated in error bars. In order to analyse differences between the curves we calculated area under curves and compared these values using a Student’s *T*-test.

### Western blots

Cells were seeded in 6-well plates and transfected at seeding as needed. Cells were seeded at 300,000 cpw in 2000 ul medium. Cells were incubated overnight and drugs (DMSO, DCT) were added in additional medium (200 ul) and were left on for required periods (24 h). Cells were washed once with PBS and lysed in buffer (50 mM TRIS ph7.5, 0.5% SDS) with cOmplete Mini preparation (Roche). Lysates were collected in Qiagen shredder columns (Qiashredder) and centrifuged through the column for 2 min at high speed. Lysates were buffered in NuPage LDS sample buffer with 5% mercaptoethanol, and heated to 100 °C for 5 min. Western blotting was performed using the NuPage system, with NuPage Bis-Tris 4–12% precast gels. Gels were run in MES buffer (NuPage MES SDS running buffer, 135 V, 80 min), and were transferred onto nitrocellulose membranes (GE Healthcare Amersham Protran 0.2NC) in NuPage transfer buffer plus 20% methanol (100 V, 90 min). Membranes were stained with Poinceau solution, washed in TBS, blocked in 5% milk in TBS for 1 h and incubated with primary antibodies (see below) at manufacturer’s recommended concentrations overnight. Blots were washed with TBS and incubated with secondary antibodies (Licor) for 1 h. Blots were washed in TBS followed by distilled water and were screened on Licor System. Licor software was used to perform quantitation.

Antibodies:

CDC20: CDC20 (D6C2Q) Rabbit mAB #14866, Cell Signaling Technology

DLGAP5: HPA005546, Sigma Aldrich

AR: AR(441):sc-7305, Santa Cruz Biotechnology

TUB: Anti-Tubulin Antibody YL1/2 ab6160, abcam

CCNB1: Anti-Cyclin B1 Antibody ab32053, abcamFKBP5: FKBP5 (D5G2) Rabbit mAB #12210, Cell Signaling Technology

GAPDH: GAPDH Rabbit PolyAB CatNo 10494-1-AB, Proteintech Europe

### ICC/IF

For immunofluorescence, LNCaP cells were plated on Greiner black myClear Cellstar 96-well plates (Greiner 655090) and were transfected with siRNA at seeding. Cells were seeded at 12,000 cpw. After incubation for 36 h, 0.5 nM DCT or DMSO was added to the wells and plates were incubated for an additional 12 h. Cells were then washed once with PBS and fixed in 4% para-formaldehyde for 20 min. Plates were washed three times in PBS, blocked for 1 h in PFS (0.5 L PBS, 3.5 g fish skin gelatin, 1.25 mL 10% saponin stock) and incubated with primary antibodies (DLGAP5, AR, TUB, same as above) in PFS at manufacturer’s recommended doses overnight. Cells were washed in PFS and incubated with secondary antibodies (Alexa 488, 594, 647, DAPI) in PFS for 1 h. After another round of washing, wells were filled with 200 ul PBS and plates were screened using the Opera system at ×20 and ×63 resolution. Images were analysed using the system software (Columbus High Content Imaging and Analysis Software, PerkinElmer). Meta- and post-metaphase cell counts were performed manually using the ×20 resolution images with Tubulin and DAPI channels (36 image fields per well, three replicate wells per condition per experiment). Ratios of post-meta/metaphase cells were calculated for each well, ratios were averaged over replicate wells in each experiment, and mean of experiments was plotted with error bars indicating SEM.

### Computational analyses

Gene expression data were downloaded from the Gene Expression Omnibus (GEO) (GSE21034, GSE16560, GSE25136, GSE35988, GSE44905, GSE60721, GSE4636, GSE69330) and from The Cancer Genome Atlas (TCGA) portal (TCGA-PRAD). TCGA, GSE21034 and GSE16560 data sets were classified using our previously described classification scheme^2^. In short, gene expression signatures characterizing ESC, iPSC, PRC2 targets, p53 status (p53 + , p53−), PTEN status (PTEN + , PTEN−), MYC status (MYC upregulated), RAS pathway, Cytokines, TMPRSS2-ERG fusion, Mesenchyme, Proneural and Proliferation, were analysed using single-sample GSEA (ssGSEA)^48^ on each sample in a data set, resulting in an array of 14 signature scores per sample. The signature score arrays were then clustered using an unsupervised clustering algorithm that applies several clustering routines and a range of cluster numbers and determines the optimal clustering using a Bayesian arbiter (best fit to a mixed Gaussian data model). In all three data sets, we found a cluster that was highly associated with the ESC, PTEN− and p53− signatures as well as with proliferation signatures (Proliferation, MYC), and a group with the opposite signals. In each of the three data sets, the differential expression of all individual genes was calculated on these groups (stem-like versus normal-like). To compare these across data sets, since not all genes were represented on all platforms, we designated a gene to be significant if it satisfied a significance cutoff in at least two data sets. For the resulting list of genes, GeneCard annotation was downloaded and searched for terms: cell cycle, PI3K, p53, DNA repair, DNA damage, stem cell, meiosis and spermatogenesis. This included a search of the literature annotations associated with each gene in addition to its descriptors. Genes were also evaluated for their expression in the cell lines PC3, DU145 and LNCaP using data from GSE21034, GSE44905 and internal RNASeq data (Hing Leung). Genes were included in the final list based on an evaluation of their *p*-values of enrichment in the data sets, their expression (present) in the cell lines, and their annotations. To evaluate the list of 48 genes as a signature, we used ssGSEA to calculate signature scores for each sample in a set, and then clustered the scores into three groups using kmeans. Survival data was then analysed using Kaplan–Meier analysis and logrank *p*-values. It was tested in a fourth cohort^6^. Mean expression of the gene set was used to test differential expression in sample types in additional gene sets^7–9^ and ANOVA *p*-values were calculated using MATLAB standard script. All data were handled using Matlab and Unix shell script.

### Statistical analyses

Screening data were analysed for quality control, and Z prime scores were calculated for each plate using the Dotmatics Studies software (all Z prime scores were greater than 0.5). Each plate contained eight samples negative and positive controls (siNTC, siAS). The median of the eight negative controls on each plate was used to calculate sample values for the siRNA probes (value = (median(siNTC) − SAMPLE) / (median(siNTC)) × 100). Mean of three experiments was calculated for each cell line, each gene, each sequence and each condition (DMSO, DCT). Window sizes were calculated across all genes and sequences in a given cell line, and mean plus two standard deviations was used as a cutoff to determine significant sequences. Genes with two or more significant sequences were considered significant in that cell line. Furthermore, combination indices (CI) were calculated using the Bliss independence model, for each gene, sequence and experiment in each cell line. For each cell line, we plotted the distribution of all CI scores and determined the 0.05 quantile in order to obtain a significance value relative to the screening data.

Incucyte data were downloaded as percent confluence in phase and green channels (CytoxGreen). Mean of technical replicates (three or more wells per condition, two images per well per scan) was used for all calculations and data were normalized by the confluency at time of drug addition for all conditions. Relative GI curves were then calculated as DCT/DMSO and plotted using mean of experiments and error of means. Significant differences between groups of curves were determined by calculating AUC using the trapezoidal rule for each curve, followed by Student’s *T*-test.

## Electronic supplementary material

Supp Figure 1

Supp Figure 2

Supp Figure 3

supplementary figure legends
