# Supplementary figures and images for: A functional genomics screen reveals a strong synergistic effect between docetaxel and the mitotic gene DLGAP5 that is mediated by the androgen receptor
Source: Cell Death Dis. 2018 Oct 19;9(11):1069. doi: 10.1038/s41419-018-1115-7 (PMC6195526; doi:10.1038/s41419-018-1115-7)

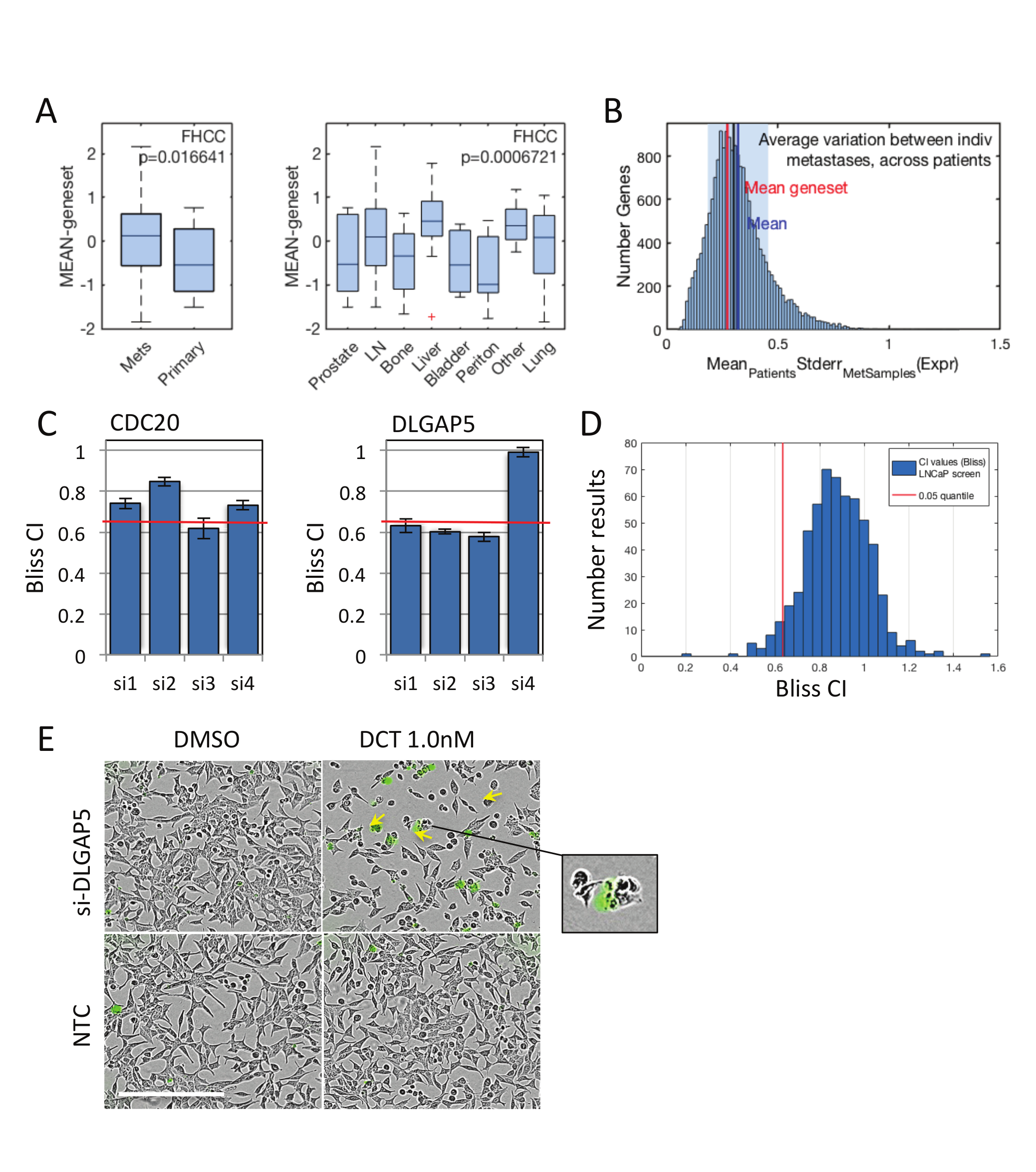

Supplement: Supplementary file 1 — Supp Figure 1 [file 41419_2018_1115_MOESM1_ESM.tif]

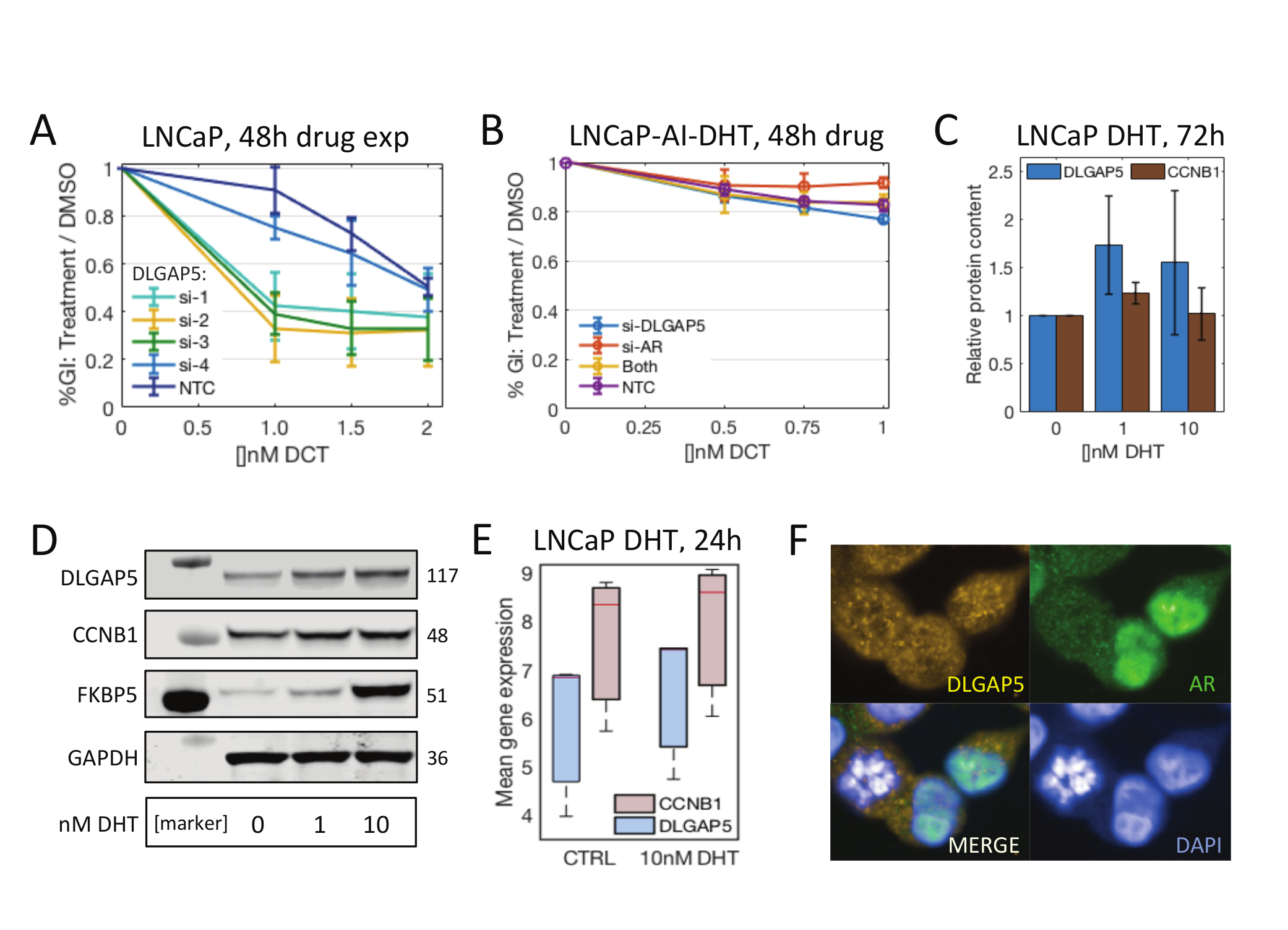

Supplement: Supplementary file 2 — Supp Figure 2 [file 41419_2018_1115_MOESM2_ESM.tif]

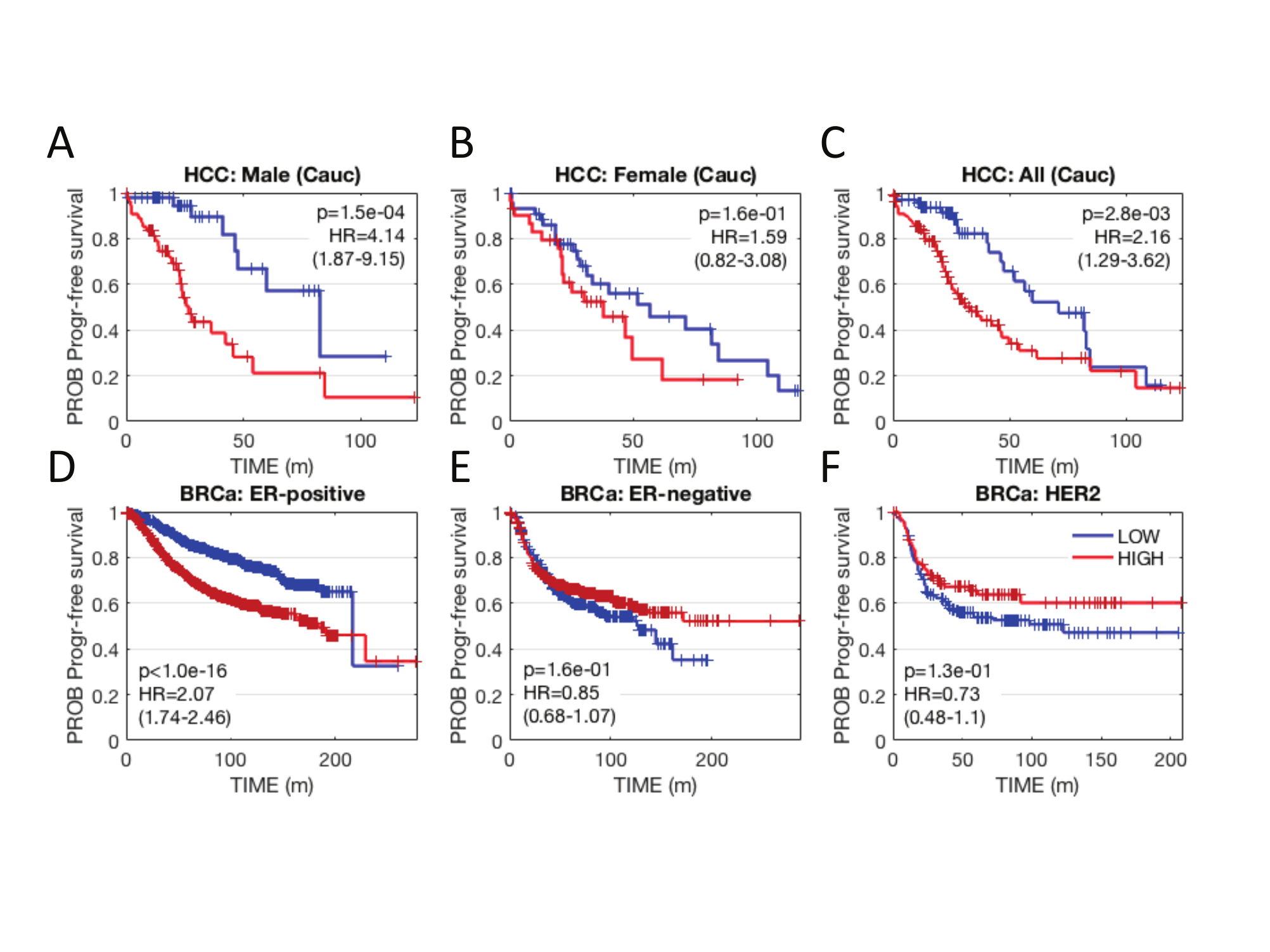

Supplement: Supplementary file 3 — Supp Figure 3 [file 41419_2018_1115_MOESM3_ESM.tif]
