# Supplementary material for: A functional genomics screen reveals a strong synergistic effect between docetaxel and the mitotic gene DLGAP5 that is mediated by the androgen receptor
Source: Cell Death Dis. 2018 Oct 19;9(11):1069. doi: 10.1038/s41419-018-1115-7 (PMC6195526; doi:10.1038/s41419-018-1115-7)
Supplement: Supplementary file 4 — supplementary figure legends [file 41419_2018_1115_MOESM4_ESM.docx]

Kay Hewit^1,3^, Emma Sandilands^1^, Rafael Sanchez Martinez^1^, Daniel James^2^, Hing Leung^1,2^, David Bryant^1^, Emma Shanks^2^ and Elke K Markert*^,1^

Title

A functional genomics screen reveals a strong synergistic effect between docetaxel and the mitotic gene DLGAP5 that is mediated by the androgen receptor.

**Supplementary Figure Legends.**

**Supp Fig 1**. A) Type analysis for Fred Hutchinson Cancer Center (FHCC) cohort. Left plot shows increased mean expression of the 48-gene set in metastatic versus primary samples, right hand side plot shows variation among samples from different tissues (met and primary). P-values were calculated by overall anova. B) In the same set, variation of expression between individual metastases from the same patient was measured for all patients and each gene. Histogram of the average variation across patients is shown, a red line marks the mean of the 48-gene set, a blue line the mean over all genes, and a black line the median of all genes. Shaded blue area marks standard deviation from the mean. C) Bliss independence model. Shown are combination indices (CI) based on the siRNA screening data in LNCaPs, for the individual siRNAs against *CDC20* (left plot) and *DLGAP5* (right plot). Red line indicates the 0.05 quantile of the distribution of all CI values for all genes and siRNAs in the LNCaP cells. D) Histogram of the distribution of CI values in the LNCaP screen. E) Images from the Incucyte assays performed in LNCaP cells. Images were taken 48h after drug addition. Yellow arrows: Green phase shows CytoxGreen activation. Scale bar, 300um.

**Supp Fig 2.** A) Dose response curve for individual siRNAs against *DLGAP5*. B) LNCaP-AI cells were grown in hormone-depleted medium and medium was replaced with full culture medium (RPMI with 10%FBS) supplemented with 10nM DHT at transfection. Dose response curves based on Incucyte growth assay. C,D) LNCaP cells were grown for 24h in androgen-depleted medium, followed by addition of DHT at 1nM or 10nM respectively. Cells were harvested after another 72h and protein contents were measured by Western blot. Quantification shows mean+stderr of n=3 experiments. E) Gene expression in LNCaP cells grown in androgen-depleted medium for up to 48h, followed by addition of 10nM DHT for 24h. Microarray gene expression data from three individual data sets (GSE60721, GSE4636, GSE69330) was collected and analysed. Box plot shows mean+stderr of n=3 reported experiments. F) ICC/IF image of mitotic cell in siDLGAP5 condition (DMSO). The AR protein is depleted during mitosis.

**Supp Fig 3**. Survival prediction based on DLGAP5 expression in different cancer types was analysed using data provided by KMplotter ([www.kmplot.com](http://www.kmplot.com)). Optimal cutoffs were calculated by the software in each setting. Insets show logrank p-values as well as odds ratio (HR, with 95% confidence intervals). A,B,C) In hepatocellular carcinoma, the predictive power of DLGAP5 expression changes with gender (n=181 total, n=102 male, n=79 female). Plotted are the subsets of male and female Caucasian patients, showing a 2.6-fold change in HR. Across all races the fold change in HR was 1.76 (n=246 male, n=118 female). D,E,F) In breast cancer, the predictive value changes with estrogen receptor (ER) status (n=2061 ER-pos, n=801 ER-neg, n=251 HER2+).
